# Slippery scales: Cost prompts, but not benefit prompts, modulate sentencing recommendations in laypeople

**DOI:** 10.1371/journal.pone.0236764

**Published:** 2020-07-31

**Authors:** Eyal Aharoni, Heather M. Kleider-Offutt, Sarah F. Brosnan, Sharlene Fernandes

**Affiliations:** 1 Department of Psychology, Georgia State University, Atlanta, GA, United States of America; 2 Department of Philosophy, Georgia State University, Atlanta, GA, United States of America; 3 Neuroscience Institute, Georgia State University, Atlanta, GA, United States of America; 4 Center for Behavioral Neuroscience, Atlanta, GA, United States of America; Universidad Loyola Andalucia Cordoba, SPAIN

## Abstract

Do people punish more than they would if the decision costs were more transparent? In two Internet-based vignette experiments, we tested whether juvenile sentencing recommendations among U.S. adults are responsive to variation in the salience of the taxpayer costs and public safety benefits of incarceration. Using a 2 Cost (present vs. absent) x 2 Benefit (present vs. absent) factorial design, Experiment 1 (N = 234) found that exposure to information about the direct costs of incarcerating the juvenile offender reduced sentencing recommendations by about 28%, but exposure to the public safety benefits had no effect on sentences. Experiment 2 (N = 301) manipulated cost-benefit salience by asking participants to generate their own list of costs of incarceration, benefits of incarceration, or an affectively neutral, unrelated word list. Results revealed a similar selective effect whereby sentencing recommendations were reduced in the cost condition relative to the benefits and control conditions, but sentences in the benefit condition did not differ from the control. This combined pattern suggests that laypeople selectively neglect to factor cost considerations into these judgments, thereby inflating their support for punishment, unless those costs are made salient. These findings contribute to the debate on transparency in sentencing.

## Introduction

When the benefits of a resource are transparent to the consumer but the costs are obscured, consumption of the resource will tend to escalate [1]. Applied to public opinion on criminal sentencing practices, scholars have theorized that such cost-benefit asymmetries could lead people to support harsher punishments than they would under more balanced conditions [2–4].

At the federal level, professional judges making sentencing decisions are generally discouraged from considering the costs of incarceration, which might include direct monetary costs, opportunity costs of foregone support for other public services, and collateral consequences for offenders, their families, and communities. Consideration of such costs has been argued to be the exclusive domain of legislators [5, 6]. But what about the voters and taxpayers whom these officials represent? If ordinary people inadvertently neglect the costs of incarceration when forming punishment attitudes, or at least discount them relative to the benefits (such as increased public safety), this behavior could systematically inflate their support for punitive measures, as expressed through voting behavior and civic discourse. To understand the degree to which ordinary people neglect to consider the costs of incarceration when forming punishment attitudes, it is instructive to examine whether increasing people’s awareness of those costs tempers their punitive attitudes. Such a finding would be useful for legislators and other legal representatives tasked to represent the diverse values of their constituents.

At least three theoretical perspectives generate predictions about the way cost-benefit exposure might affect lay punishment judgments. First, from a deontological perspective, exposure to information about the cost of incarceration should not affect punishment judgments. In this view, sentencing judgments should be based solely on the perpetrator’s level of deservingness, not on the potential consequences [7]. In other words, the deontic moral principles driving punishment are not “for sale.” Evidence for this prediction is supported by research on sacred values, that certain types of values are absolute and unmoved by factors like market pricing [8].

Second, from a classical, rational economic perspective, in contrast, rational actors like voters and taxpayers might have self-interested reasons to factor information about the costs and benefits of the punishment into their decision. According to this rational choice perspective, as the costs of a given punishment increase, support for that punishment should decrease, and the reverse is true for the benefits. A central tenet of this perspective is that, above all, rational actors value cost and benefit information. If the benefits of a resource are more transparent than the costs, consumption of the resource will tend to increase [1, 2, 4]. By inference, if the benefits of incarceration are more transparent than the costs, people might punish more harshly than they would under more balanced conditions. Moreover, rational actors should update their prior valuation of the resource in light of new cost-benefit information. So, if they come to learn that the cost of incarceration is substantially higher than they had believed, they might reduce their support for it even though the true cost remained unchanged. Rational actors’ preferences should also be consistent across broader contexts. That is, they should not be affected by contextual factors that are exogenous to the relevant informational domain. By implication, being asked to contemplate the existence of costs of incarceration—without being provided new cost information—should not be sufficient to reduce one’s support for it.

Third, from a cognitive perspective, though people might place personal value on information about the costs and benefits of a resource, their expressed preferences for that resource may be influenced implicitly, by contextual factors outside the relevant informational domain. Once such contextual factor is how salient, or conspicuous, that information is, irrespective of its informational content. Marketers, for instance, have long understood how to manipulate consumers’ preferences by making the benefits of the product—such as a stronger build—more salient than the costs, which are often relegated to the fine print. Such strategies exploit the human tendency to only consider choice options that are most immediately available in memory (i.e., the Availability Heuristics; Tversky & Kahneman, 1973 [9]). Consistent with this interpretation, research has shown, across a wide variety of applications, that people tend to neglect to consider the other potential uses of their resources unless those other alternatives are made explicit [10–14]. It follows that if the benefits of incarceration are more psychologically salient than the costs, then ordinary people, including voters and taxpayers, might tend to support the use of harsher punishment than they would if the costs and benefits were equally salient.

Consistent with this prediction, a growing body of evidence suggests that exposure to a decision’s resource constraints can reduce support for criminal justice programs or policies. For instance, in traditional public opinion polls, stated support for tough-on-crime policies has historically been high [15], but when respondents are asked to tradeoff between building more prisons and expanding other costly programs like drug treatment, probation and preventive interventions, their support for prisons ranks relatively low [15, 16], suggesting that the salience of competing choice alternatives changes people’s expressed preferences.

Other studies have shown that direct cost information also reduces support for punishment. In one early study, Thomson and Ragona found that, compared to no cost information, exposing participants to the material costs of different criminal sanctions reduced support for prison relative to community service [17]. In a more recent study, participants evaluated a proposition to eliminate the use of prison for low-level offenses. The results showed that emphasizing the costs of incarceration versus the benefits predictably increased support for sentencing reform [18]. In a survey study of professional judges, exposure to information about the typical direct cost of incarceration yielded lighter sentences compared to when that cost was not provided [19], suggesting that the effect might not just be the result of a lack of legal knowledge or training.

In all of these studies, cost information had a predictable impact, but there is no way to know which judgment—with or without cost information—was more representative of people’s genuine preferences. Are people erroneously discounting cost information when it’s absent, or are they over-weighting it when it’s present? To address this question, we previously conducted a pair of vignette-based experiments, asking participants to make sentencing recommendations about an aggravated robbery and home invasion either with incarceration cost information, without cost information, or under the stipulation that the punishment would be cost-free to taxpayers (because the costs would be paid by a third party). First, we replicated the finding that exposure to information about the direct cost of incarceration reduced sentence recommendations relative to conditions with no cost information. But when no cost information was presented, sentencing recommendations were no different from those made under cost-free conditions. To the extent the cost-free condition captures people’s free punishment choices absent any self-interested motivations, then the overall pattern suggests that when cost information is not provided at all, people who would otherwise factor that information into their judgments punish as if cost is not a factor [20, 21]. In other words, cost information does not provoke people to punish less than they intend; rather, people genuinely value the cost information, but without explicit prompting, they neglect to consider it.

Together, these studies suggest that laypeople, and even legal experts, will consider the decision costs in their punishment judgments, but they do so inconsistently. That is, they neglect to factor cost considerations into these judgments unless those costs are transparent. Colloquially, they appear to invoke a heuristic that “what you see is all there is” [22]. If so, this tendency would obscure stakeholder’s genuine sentencing preferences and could generate over-consumption of criminal justice resources.

Although the existing research has generated important insights into the effects of cost salience and cost discounting on punishment judgments, less attention has been paid to the specificity of these effects. First, do people place equal consideration on sentencing costs and benefits, or is one more influential than the other? From a legal perspective, the benefits of incarceration (e.g., deterrence and incapacitation) should play a central role [23]. But among laypeople, research suggests that the extent to which such benefits guide punishment attitude formation is relatively weak [24, 25]. Moreover, research is lacking on the extent to which support for incarceration is increased by benefit prompts (but see [18]) or the extent to which such effects are counteracted by cost prompts. If punishment recommendations are more responsive to cost prompts than benefits prompts, for instance, then from a rational choice perspective, this would suggest that the cost prompt is more informative with respect to the participant’s prior knowledge.

Second, if punishment judgments are responsive to exposure to the expected consequences of incarceration, is this a calculated response to learning information that was not previously known, or will people change their judgments as a result of cost-benefit salience alone, even when no new information is provided? Thus far, the aforementioned research has assumed a necessary role for cost *information*, typically including dollar values as a part of their stimulus prompts. If new cost or benefit information is necessary to persuade people to change their support for incarceration, this would be consistent with the rational choice perspective. But if people change their punishment judgments in response to prompts containing no new information (e.g., excluding dollar values and other costs), this would suggest that heuristic processes guide typical punishment judgments. According to this cognitive perspective, if people are simply reminded that incarceration is costly or beneficial, but are not provided with any examples or amounts, and they still change their punishment judgments, then this shift is likely based on psychological mechanisms of decision making (e.g. the availability heuristic).

Finally, existing research on cost neglect in punishment judgments has disregarded the juvenile justice domain. One line of research indicates that public support for financing juvenile incarceration tends to be relatively low, but in these studies, the putative cost of incarceration was always explicit [26, 27], precluding a test of cost salience. Examining punishment cost neglect in the juvenile context is pertinent because juvenile incarceration costs are typically far greater (about five times greater) than those of adults [28] To the degree that lay punishers value cost considerations but lack the subject-matter knowledge, we expect that their punishment recommendations will be at least as responsive to cost prompts in the juvenile context as has been observed in adult contexts, but this prediction remains to be tested.

This article reports two general-population survey experiments: (1) examining the independent contributions of provided cost and benefit information to punishment judgments for juvenile offenders, and (2) examining the effect of cost and benefit prompting on such judgments, but without providing any additional information about the costs or benefits. Human subject research was authorized by the Georgia State University institutional review board: H16349. Written consent was obtained from all participants. All study data files are available from the Open Science Framework (DOI 10.17605/OSF.IO/UVNFY).

We predicted that, under both of these conditions, increased cost salience would drive down sentencing recommendations because, while people care about the costs, previous research supports that they do not spontaneously consider them without prompting. The inverse effect was not predicted for increasing benefit salience. Although people often explicitly justify their support for incarceration in terms of benefits like deterrence and incarceration, implicit measures of punishment attitudes casts doubt on their actual motivational influence [24, 25]. Moreover, to the extent that people do value such benefits of incarceration, we suspect that such benefits are already a salient feature of criminal punishment decisions, so any new benefit information presented on this topic could have limited marginal effect. Evidence of a selective effect of cost salience on juvenile punishment judgments is important because it would suggest the operation of a systematic bias in how laypeople, including voters and taxpayers, weigh the costs versus benefits of these punishment judgments.

## Experiment 1

The purpose of this experiment was to test the specificity of the effect of cost salience on punishment judgments in a juvenile justice context. Toward this end, we employed an experimental vignette method wherein participants made sentencing recommendations about individual offenders. Although laypeople do not render sentencing recommendations in juvenile cases, we made this design choice because, compared to more abstract policy-position approaches, case narratives about individual offenders are likely to provide a concrete way of activating people’s genuine punishment attitudes. If sensitivity to cost-benefit information is the result of a calculated response to new information, the effect should be bidirectional, such that exposure to the costs evokes punishment decreases, exposure to the benefits evokes punishment increases, and when both cues are present, any cost and benefit effects should, to some degree, cancel each other out. But if people do not value typical benefits of incarceration, or neglect to consider them by default (i.e., without exposure), then the marginal effect of additional benefits exposure on punishments should be relatively weak; Cost exposure, thus, would exert a disproportionate effect on such punishments. A supplemental aim of this experiment was to examine, in an exploratory fashion, whether any observed effects were influenced by participants’ self-reported political ideology, socioeconomic status (SES), or emotion regulation abilities, or their explicit justifications for punishment (i.e., where retributivists less affected by cost prompting?).

### Method

#### Participants

Participants were 297 U.S. adults recruited on Amazon Mechanical Turk (see [29]) and paid $2.00 for their participation. Participation was restricted by age (18+ yrs.), country (U.S.), and an approval rating of 96% or higher (see [30]). Individuals who had previously participated in Mechanical Turk surveys by the principal investigator were blocked from participation. Eighteen were excluded for incomplete data; 11 for failing a multiple-choice attention check (“What are the colors of the American flag?”); 23 for failing to recognize the correct crime type from a multiple-choice list; and 11 for failing to recognize the cost condition to which they were exposed. The remaining 234 reportedly were 50.0% male, 48.7% female, and 1.3% “other” or “prefer not to answer”; 13.2% Hispanic or Latino; 76.5% White/Caucasian, 13.2% Black or African American, 7.3% Asian, and 4.7% other/unknown (ethnic and racial categories were non-exclusive); and with a mean age of 36.4 (*SD* = 11.7). Numbers of participants were similar across conditions, as were gender ratios: Cost Absent (n = 117, 50.4% M), Cost Present (n = 114, 50.9% M), Benefit Absent (n = 113; 52.2% M), Benefit Present (n = 118; 49.2% M).

#### Design and hypotheses

The study design was a 2 Cost (present vs. absent) x 2 Benefit (present vs. absent) between-subjects factorial design with random assignment to conditions. In the present-present condition, the order of the cost and benefit statements were counterbalanced. The dependent measure was a sentencing judgment about time in juvenile detention.

***H1*.** We hypothesized a main effect of cost salience such that sentencing recommendations would be lower when cost information was present than absent. We did not predict a change in punishment as a function of benefit salience or the cost-by-benefit interaction because we expected that people would have already factored the benefit information into their initial punishment judgments by default.

#### Materials and procedure

The criminal case summary was adapted from Rachlinski et al. [19] and described a fictitious juvenile defendant convicted of drug trafficking, followed by statements putatively provided by the sentencing advisory commission. The statement of the benefits of incarceration reported that incarcerating the defendant would likely prevent three new violent crimes for each year in custody because his detainment would reduce the number of unlawful debt collections and other risky encounters in the criminal supply chain. (The stipulation of “three” violent crimes was empirically derived from a pilot test of 32 independent Mechanical Turk workers responding to the multiple choice question: “How many violent crimes do you think are actually prevented when a drug trafficker is incarcerated for 1 year in prison in the United State on average?”.)

The statement of the costs of incarceration reported the true-to-life taxpayer cost of $148,000 for each year of custody [28]. Defining the cost of incarceration in terms of direct monetary cost is admittedly narrow and ignores the many other potential collateral consequences of incarceration. To offset this problem, we specified that the funds spent on incarceration is money that could otherwise have been invested in reentry support services known to reduce the risk of reoffending. This design choice means that it is not possible to determine which of these two cost types is the primary driver of any observed effects, but if they operate in combination, this would be consistent with our theoretical framework. The crime of drug trafficking was used because it is a common example of a crime that is relatively moderate in seriousness, and while an explicit test of the effect of crime seriousness was beyond the scope of this project, we nonetheless expect that sensitivity to cost information will be greatest among crimes of low to moderate seriousness. The vignette text and manipulations were as follows:

Joseph Campbell, a high school dropout, was arrested at a party for allegedly selling 50 grams of methamphetamine. Joseph was charged with drug trafficking. The evidence at trial, which included testimony from an undercover police officer and two other witnesses, showed convincingly that he exchanged the methamphetamine for $3,000 in cash. Joseph is 17-years-old, has a spotty employment record, and a history of drug addiction. He has one prior conviction for possession of methamphetamine.

In your jurisdiction, methamphetamine sales carries a maximum sentence of 6 years in a secure juvenile detention center. [BENEFIT STATEMENT:] According to your jurisdiction's sentencing advisory commission, incarcerating Joseph would likely prevent 3 new violent crimes for each year of custody. This is because his detainment would reduce the number of unlawful debt collections and other risky encounters in the criminal supply chain. On the other hand, [COST STATEMENT:] the commission states that incarcerating Joseph would likely cost taxpayers $148,000 for each year of custody. This is money that could otherwise have been invested in reentry support services known to reduce the risk of reoffending.

Immediately following the narrative, the dependent measure was delivered. Participants were asked to indicate how much time in a juvenile detention center the defendant should receive on a ratio slider scale from 0 to 6 years. We also assessed participants’ relative endorsement of five common motivations for punishment [31] to test whether people who are motivated by retribution might be less responsive to cost salience than others. This instrument asks respondents to rank order statements describing one of four justifications for punishment: retribution, specific deterrence, general deterrence, and rehabilitation (e.g., “People who commit crimes should be punished because: [RETRIBUTION] by punishing them we give them what they deserve and giving offenders their just deserts is a good thing”). Respondents who ranked the retributive item among their top two justifications were classified as relatively supportive of retributivism. All others were classified as more supportive of utilitarianism. The Difficulties in Emotion Regulation Scale (DERS-SF [32]) was also collected to explore its relation to cost salience, predicated on a significant association between DERS total score and punishment recommendations. Finally, we assessed participants’ self-reported demographic information, including political ideology, from very liberal (-3) to very conservative (+3), and SES (lower, lower-middle, upper-middle, and upper).

#### Results

As a check of the credibility of the evidence, participants were asked whether there was enough evidence to support the defendant’s conviction. The vast majority (94.83%) answered affirmatively.

Next, a two-way Analysis of Variance (ANOVA) was employed to test for any independent and interactive effects of cost and benefit information on recommended sentence length. (A pre-test showed no main effect of order on sentencing recommendations within the present-present condition, *F*(1, 57) = .09, *p* = .771, so we did not enter order as a factor in our models.) The overall model was significant, *F*(3, 230) = 5.02, *p* = .002, 1-β = .91, and all comparisons were consistent with our hypothesis. First, we found a main effect of cost, *F*(1, 230) = 13.58, *p* < .001, 1-β = .96, such that sentencing recommendations were significantly (~28.7%) lower in the presence of information about the cost of incarceration (*M* = 1.96, *SE* = .15, 95% CI [1.66, 2.26]) than without it (*M* = 2.75, *SE* = .15, 95% CI [2.45, 3.04], η^2^ = .056, Bonferroni corrected). Yet, there were no main effects of benefit information, *F*(1, 230) = .89, *p* = .348, 1-β = .16, (*M-*present = 2.25, *SE* = .15; *M*-absent = 2.45, *SE* = .15), and no cost-by-benefit interaction, *F*(1, 230) = .51, *p* = .478, 1-β = .11.

**Fig 1:**
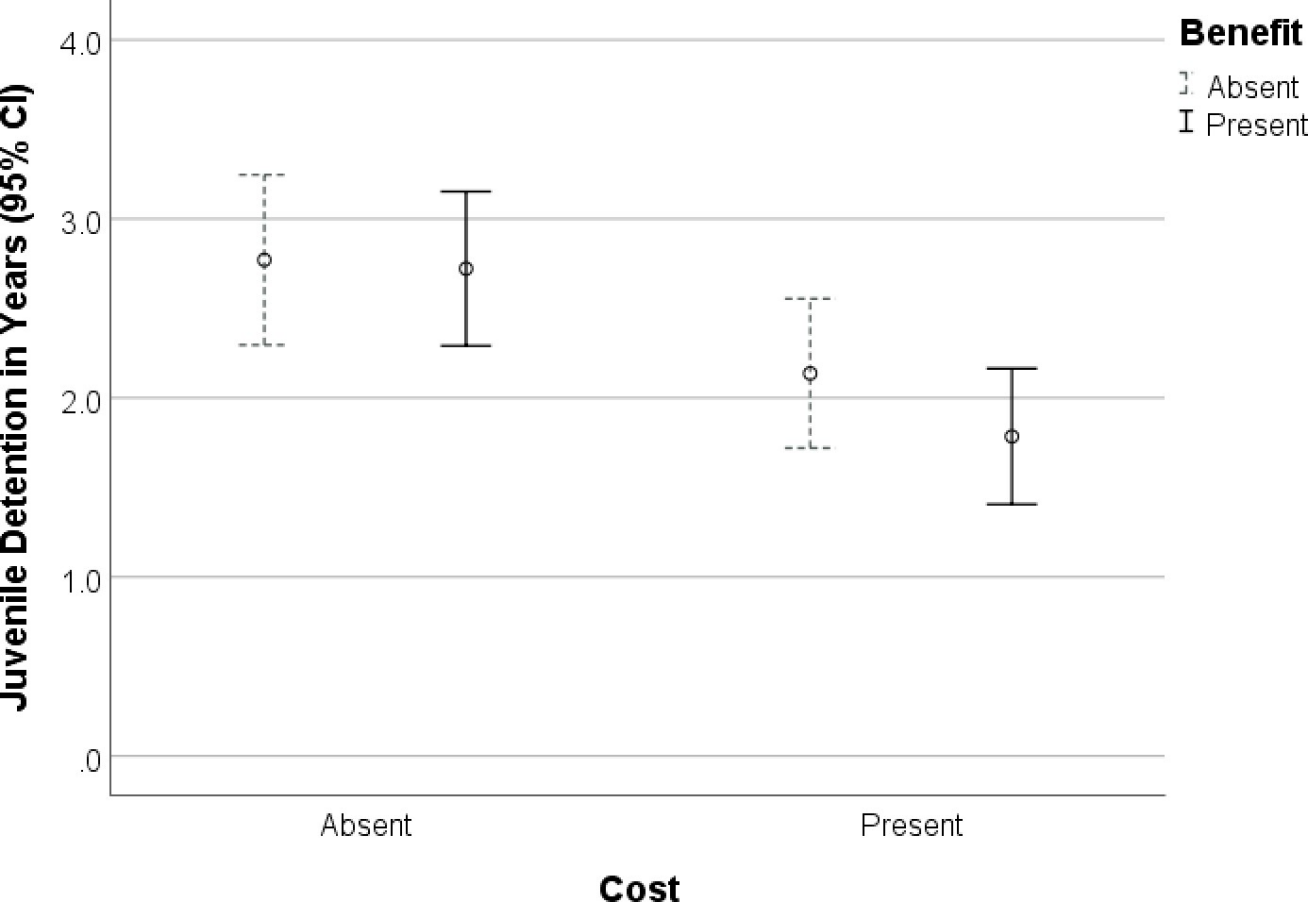
Sentence recommendations were lower following exposure to information about the cost of incarceration relative to no cost information (*p* < .001), but sentence recommendations with and without exposure to benefit information did not differ from one another (*p* = .348), nor was there a cost-by-benefit interaction (*p* = .478).

To more fully evaluate the effect of cost information on sentences, we constructed a hierarchical linear regression to rule out possible influences of age and gender. Age and gender were entered as first-level predictors, and the cost condition was entered at the second-level. Neither age nor gender exerted main effects on sentence length, *R*^2^ = .009, *F*(2, 201) = .90, *p* = .409, but cost information exerted the predicted effect above and beyond age and gender, *R*^2^ = .044, *t*(201) = -2.71, *p* = .007, B = -.618, η_p_^2^ = -.188. Taken together, punishment judgments were selectively responsive to cost information. Presenting benefit information did nothing to counteract this effect (See Fig 1).

Next, we asked whether people who ranked retribution as an important justification for criminal punishment might be less responsive to the reductive effect of cost salience on punishment than those who ranked retribution as less important. According to a two-way ANOVA with cost level (present vs. absent) and punishment justification (support for retributivism vs. utilitarianism) as between-subjects factors, there was not a significant interaction between support for retribution and cost salience on sentencing recommendation, *F*(1, 230) = .97, *p* = .33, 1-β = .16, suggesting that sensitivity to cost salience might have similar effects on individuals regardless of their explicit justifications for punishment. However, descriptively speaking, the mean scores demonstrated consistency with the predictions of such an interaction, whereby participants who were exposed to cost information and who ranked low in the retributive motive tended to recommend lighter sentences than those in the other three conditions (retributive/cost present *M* = 2.86; *SE* = .49, 95% CI [1.90, 3.83]; retributive/cost absent *M* = 2.98, *SE* = .54, 95% CI [1.91, 4.04]; non-retributive/cost absent *M* = 2.73, *SE* = .16, 95% CI [2.42, 3.03]). Thus, it is possible that this model was not sufficiently powerful to detect a true interactive effect.

Last, we separately examined whether the effect of cost salience on sentencing recommendations might depend on self-reported political ideology or SES (characterized by a median split), or on the total score of the Difficulties in Emotion Regulation Scale (DERS). A two-way ANOVA with political ideology yielded a significant overall model, *F*(3, 228) = 8.07, *p* < .001, 1-β = .99, with a marginal interaction, *F*(1, 228) = 3.18, *p* < .076, 1-β = .43, suggesting that, relative to no cost information (*M* = 2.65, *SE* = .21, 95% CI [2.23, 3.08]), the presence of cost information tended to reduce sentencing recommendations for liberals (*M* = 1.50, *SE* = .21, 95% CI [1.09, 1.92], *p* < .001) but not conservatives (*M* = 2.84, *SE* = .21, 95% CI [2.44, 3.25], *p* = .18). SES, however, did not moderate the effect of cost salience on sentence recommendations, *F*(1, 219) = 2.29, *p* = .13, 1-β = .34. Finally, DERS score was not correlated with sentencing scores (*r* = .02, *p* = .749), so no further analysis of this question was conducted.

The overall pattern of results suggests that juvenile punishment recommendations are predictably responsive to exposure to information about the costs of incarceration but not the benefits. The cost effect discredits the deontological prediction that punishment judgments should exclude cost considerations. In contrast, the cost effect can be explained by the rational choice perspective, which predicted that people should factor new cost information into their punishment judgments. From this perspective, the cost effect permits the inference that participants, on average, valued the costs of incarceration, and the cost information provided was significantly greater than any prior estimate they might have consulted.

The null effect of the benefit information in this first experiment is open to interpretation. This result could potentially indicate that our participants did not value deterrence, but this interpretation would directly contradict a body of research that suggests otherwise (e.g., [33–35]), as well as our own pilot data. A more plausible explanation, still consistent with the economic framework, is that the benefit information provided was not ultimately informative to participants because the deterrent effect, as we defined it, was on par with what participants already believed. That is, it did not require them to update their prior beliefs, so an increase in punishment was not necessary. While this rational choice perspective is plausible, the cognitive perspective made similar predictions. Experiment 2 was constructed to de-confound these alternative explanations.

## Experiment 2

Extending the findings of Experiment 1, Experiment 2 tested whether the reductive effect of cost exposure on punishment is a calculated response to learning new information relevant to the decision, or instead the result of cognitive availability, whereby a minimal prompt about the presence of those costs is sufficient to inspire preference change, even without new information being presented.

The rational choice perspective is predicated on the introduction of new cost-benefit information and predicts consistency across exogenous contexts. So, if sentencing attitudes are truly driven by economic reasoning, then these attitudes should not be influenced by mere appeals to prior beliefs about the costs and benefits of incarceration. But if sentencing judgments change in response to self-generated cost-benefit information, even when no new information is provided, this would violate the consistency tenet of the rational choice perspective and instead favor the cognitive perspective. According to this perspective, though people may ultimately care about the costs of incarceration, they do not spontaneously consider those costs without the aid of external prompts that make these costs more salient. This prediction formed the basis of Experiment 2.

### Method

#### Participants

Participants were 343 U.S. adults recruited on Amazon Mechanical Turk. All recruitment procedures were the same as Experiment 1 except that they were paid $1.50 for their participation. Twenty-one were excluded for incomplete data; 7 for failing the “American flag” attention check; and 14 were excluded for failing to recognize the correct crime type from a multiple-choice list. The remaining 301 reportedly were 57.1% male, 42.5% female, and 0.3% preferred not to answer; 11.0% Hispanic or Latino; 81.1% White/Caucasian, 9.0% Black or African American, 7.0% Asian, and 5.3% other/unknown (ethnic and racial categories were non-exclusive); and with a mean age of 37.7 (*SD* = 12.4). Numbers of participants were similar across conditions: Salient Cost (n = 105, 65.7% M), Salient Benefit (n = 97, 48.5% M), Control condition (n = 99; 56.6% M).

*Design and Hypotheses*. The experiment employed a between-subjects, three groups design with random assignment to one of three prompts: salient cost, salient benefit, and a control condition. A cost-and-benefit condition was not included in this design because no such interaction was observed in Experiment 1. All participants read a hypothetical criminal case summary about a juvenile drug trafficking case. The two treatment conditions prompted participants to generate three examples of costs and benefits of incarceration, respectively. The control condition prompted participants to generate a list of three unrelated words. This condition enables us to understand how the cost and benefit conditions compare, not just to each other, but to a thematically neutral benchmark designed to take about the same amount of time. We limited each list to three items to minimize demand characteristics. Specifically, research on the availability heuristic suggests that when people are unable to generate a large number of requested items, they tend to interpret their relatively low performance as evidence that the real world must not contain many instances of that item [36]. The dependent measure was a sentencing judgment.

***H1*.** Our primary hypothesis was that, when participants were minimally prompted (without the provision of new cost information) to consider the negative consequences of incarceration, sentencing judgments would be more lenient than in either the control or the salient benefit conditions.

***H2*.** Based on the results of Experiment 1, we also predicted that sentencing judgments in the salient benefit condition would not differ from the control condition.

#### Materials and procedure

We presented a vignette describing a fictitious case of juvenile drug trafficking. The vignette was identical to that of Experiment 1 with two exceptions: (1) The new vignette included an additional clause clarifying that the defendant was tried as a juvenile for a felony; (2) Instead of positing substantive cost and benefit information, the following manipulation was administered: In the salient cost condition, participants were asked to imagine that they had just read a newly released government report stating that custodial sentences produce many [negative] consequences for the community. They were then instructed to list three examples of possible [negative] consequences of incarcerating the defendant. Participants in the salient benefit condition followed the same instructions for positive consequences. This experiment also included an affectively neutral condition to control for the additional cognitive demands evoked by the treatment conditions, such that those who were not exposed to cost or benefit prompts would engage in an unrelated task of similar difficulty. In the control condition, participants were instructed to list three words whose third character is the letter ‘k’ (adapted from Tversky & Kahneman, 1973). (See S1 Appendix for exact vignette text and manipulations.)

Following the salience manipulation, the dependent measure was delivered. Participants were asked to indicate how much time in a juvenile detention center the defendant should receive on a ratio slider scale from 0 to 4 years, slightly shorter than the scale used in Experiment 1 to approximate a more normal distribution. All additional survey questions were the same as in Experiment 1 except that the DERS was not included, and the punishment justification scale included an additional statement for ranking the importance of giving the victim’s friends and family the revenge they deserve (coded as a retributive item).

Text-based responses to the listing task were coded for qualitative analysis. All such responses were coded by two independent, trained raters who were blind to the study hypotheses. Coding categories were predefined by the investigators. The initial categories for positive consequences were: Incapacitation, Specific deterrence, General deterrence, Rehabilitation, Justice, and Revenge. The initial categories for negative consequences were: Increased recidivism; Monetary/Opportunity costs, Collateral consequences for defendant, Collateral consequences for defendant’s family, and Violates principles of fairness/justice. A miscellaneous category was also included. Interrater reliability was assessed using all responses except those assigned by either rater to the miscellaneous category and exceeded conventional criteria (Kappa = .828). Coding discrepancies between raters were then resolved by the principal investigator where justified by our a priori category definitions. Finally, to increase representation of low frequency categories, the categories were aggregated into the following scheme: Incapacitation/Deterrence, Rehabilitation, and Retribution (for the positive consequences condition), and Collateral consequences for the offender and family, Collateral consequences for the community, and Monetary/opportunity costs (for the negative consequences condition).

#### Results

**Fig 2:**
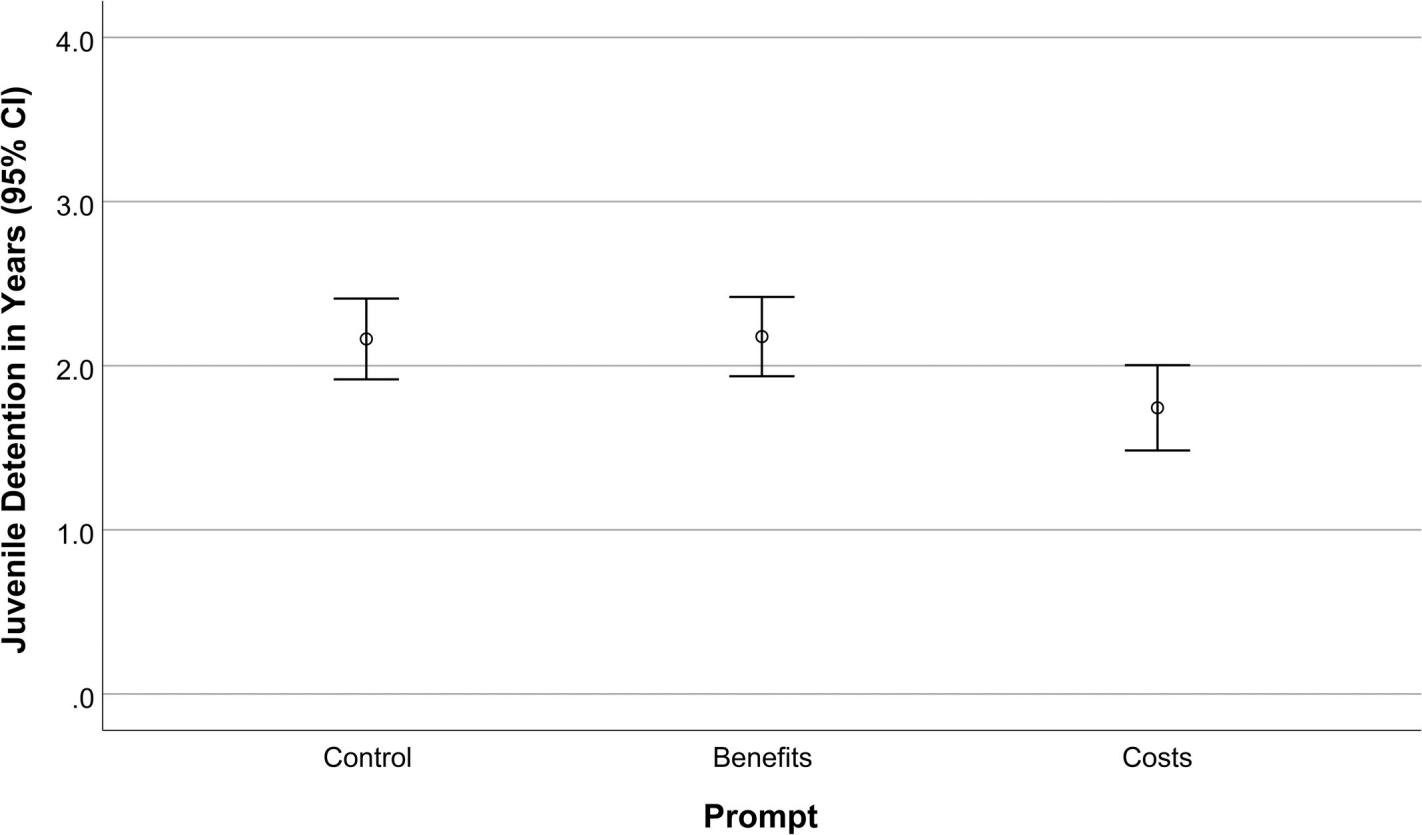
Sentence recommendations were lower following the cost prompt relative to the control prompt (*p* < .05) and benefit prompt (*p* < .05), but the control and benefit conditions did not differ from one another (*p* = .996).

Our hypotheses were tested using a one-way ANOVA with prompt (salient costs, salient benefits, vs. the control condition) as the independent variable and sentence recommendation as the dependent variable. The model was significant, *F*(2, 298) = 3.81, *p* = .023, 1-β = .69. As predicted, participants who were prompted to generate a list of negative consequences of incarceration recommended significantly (~20%) lower sentences (*M* = 1.74; *SE* = .13, 95% CI [1.49, 1.99]) than those prompted to generate a list of positive benefits of incarceration (*M* = 2.18; *SE* = .13, 95% CI [1.93, 2.43], *p* = .043, η^2^ = .019) and those in the control condition (*M* = 2.16; *SE* = .12, 95% CI [1.92, 2.41], *p* = .047, η^2^ = .019), but sentences across the latter two conditions did not differ, *p* = .996, suggesting a cost-specific effect, using Tukey’s HSD correction for multiple comparisons (See Fig 2).

To rule out any possible influence of age and gender, we constructed a hierarchical linear regression with age and gender as first-level predictors, and prompt at the second level. As in Experiment 1, age and gender did not exert main effects on sentence recommendations, *R*^2^ = .002, *F*(2, 281) = .29, *p* = .750, but the prompt type retained the predicted effect controlling for age and gender, *R*^2^ = .02, *t*(281) = -2.32, *p* = .021, B = -.218, η_p_^2^ = -.138.

Since the control condition prompted participants to generate single words only, it is possible that differences between treatment and control conditions could be attributed to differences in effort. As a proxy for effort, we constructed a one-way ANOVA to test for differences in response time across the three prompt types, but none were found, *F*(2, 298) = 1.56, *p* = .212, *M* = 193.56 s, *SE* = 9.10, 95% CI [175.66, 211.47].

As in Experiment 1, we used a series of two-way ANOVAs to separately examine whether the effect of cost salience on sentencing recommendations might depend on self-reported retributive punishment attitudes, political ideology, or SES. However, cost salience did not interact with any of these variables, *F*(2, 295) = .25, *p* = .781, 1-β = .09, *F*(2, 292) = .21, *p* = .811, 1-β = .08, and SES, *F*(2, 292) = .15, *p* = .862, 1-β = .07, respectively.

As a supplemental test of the role of retributive attitudes on punishment recommendations, we explored this relationship using participants’ free-text responses about the costs and benefits of incarceration. First, we asked whether, by isolating participants who listed a retributive benefit of incarceration, we would observe an increase in punishment relative to those in the control condition. However, the small number of participants in this group precluded its analysis (n = 12), which itself suggests that most people might not tend to think of retribution as a benefit of incarceration. Next, we conducted the inverse test—isolating those who listed only non-retributive benefits—in search of a punishment decrease in this group relative to control participants. However, the overall pattern remained unchanged. That is, no difference in punishment was observed between those who listed non-retributive benefits (*M* = 1.92; *SE* = .09, n = 184) and controls, (*M* = 2.16; *SE* = .12, n = 105), *t*(287) = -1.55, *p* = .122.

As a test of our supposition that costs of incarceration are naturally less salient to decision makers than the benefits, we tabulated the frequency percentage of two types of text-based responses to the listing task: monetary/opportunity cost relative to other costs, and reduced recidivism relative to other benefits. This choice corresponds to the way costs and benefits were operationalized in the criminal case summary. Of all three benefit types, incapacitation/deterrence ranked first (e.g., “The offender can't re-offend while incarcerated.”), representing 56.00% of all benefits listed. The others were Rehabilitation at 39.20% (e.g., “Maybe help rehabilitate the offender.”) and Retribution at 4.80% (e.g., “It punishes him for his wrongdoing”). Descriptively, this pattern suggests a clear, collective perception that incarceration serves a recidivism reduction function. Among the Cost observations, Collateral consequences for the offender ranked first at 49.78% (e.g., “Time spent in prison will likely cause psychological damage”), followed by Collateral consequences for the community at 25.76% (e.g., “Increases chances of violent crimes occurring when defendant is released”). Monetary/opportunity costs ranked last at 24.45% (e.g., “It puts taxpayer money to waste”). This pattern suggests that monetary and opportunity costs are not the most salient of the various potential costs of incarceration.

## Discussion

The purpose of this study was to examine the effects of decision cost and benefit salience on sentencing attitudes of lay adults about juvenile offenders. We reasoned, based on our earlier work [20, 21], that when people formulate punishment judgments, they neglect to consider the costs unless explicitly prompted, but do not neglect the benefits. As a result, we predicted that prompting people to think about the benefits should not increase their sentencing recommendations, but prompting them to think about the costs should reduce these recommendations. These predictions were fully supported. Experiment 1 showed that punishments were predictably responsive to information provided about the costs of incarceration, but not the benefits. Experiment 2 demonstrated an equivalent effect using a minimal prompting manipulation, absent any new cost or benefit information. This pattern suggests that the people in our samples value the costs of incarceration but uniquely neglect to consult those considerations unless actively prompted.

These findings contradict the prediction that punishers will defy cost considerations via deontological commitments to sacred values [8]. Despite ample research suggesting that retribution is the primary driver of punishment attitudes is retribution (e.g., [24, 25, 37]), the fact that our participants rendered more lenient judgments following cost prompting suggests, at the least, a boundary condition to the predictions of sacred values reasoning.

Rational choice theory also has difficulty explaining our overall pattern of results. Although economic considerations could account for reductions in punishment in response to new cost information (Experiment 1), they cannot easily explain why this pattern persisted when participants were prompted to generate their own examples of costs of incarceration (Experiment 2). Indeed, our Experiment 2 participants violated a central tenet of rational choice theory, which states that an individual’s choices should track the relevant cost-benefit information but should be robust to changes to the external context. This effect, however, is a direct prediction of the cognitive perspective, which suggests that people’s expressed preferences for a given resource may be unwittingly influenced by the degree to which the relevant costs or benefits of that resource are cognitively available.

Our pattern of results aligns closely with other research, including studies of sentencing attitudes [18–21, 38], showing that cost prompts can induce people to trade off these so-called sacred values [39]. The present results extend these previous findings by demonstrating that the effect of cost-benefit salience on punishment judgments may be specific to costs and may represent an implicit effect of cognitive availability, as opposed to a calculated response to gaining new insight about the costs of incarceration.

This study is also the first to demonstrate an effect of cost salience on sentencing attitudes toward juvenile offenders. We expected attitudes toward juvenile offenders to be at least as responsive to cost salience as it is toward adults because of the high cost of juvenile detention [28] and because of general societal support for juvenile rehabilitation. Ultimately, the observed decreases in punishment were on par with that of our 2019 study involving adult offenders (between 20–30%), but note that the sentencing scale range varied between these two studies, as it does in real adult versus juvenile sentencing policies.

The question remains regarding why people’s punishment judgments might shift in response to cost prompts but not benefit prompts. One possibility, defended elsewhere, is that people don’t really care very much about the benefits [24, 25]. We offer a different interpretation—that such cost-benefit asymmetries might reflect a more general bias in how individuals manage multiple, sometimes conflicting motivations, such as the affirmative motivation to get justice or to increase public safety versus the competing motivation to invest in other valued social services (see also [39]). Whether prompted by intrinsic psychological cues or extrinsic structural cues, the dominant motivation will selectively bias how the individual weighs new incoming information. Critically, it will tend to place a premium on the decision’s expected benefits (with respect to that motivation) relative to the costs because realizing the benefits is definitional of goal fulfillment. In the context of criminal sentencing, for example, the whole point of the sentencing hearing is to decide, not how much money to save taxpayers, but how much the offender should be *punished*. This “benefit” framing treats the costs as exogenous to the fulfillment of the goal. If anything, they are obstacles to the goal, not goals in their own right. The individual may thus discount those costs, or neglect them entirely (see also [22, 40]).

But in a more fundamental way, costs do represent goals. They represent the potential benefits of the other alternative options that would be forgone by fulfilling the more salient goal. So in our studies, the fact that experimentally increasing the salience of those neglected opportunities increases their decision share confirms the proposition that these neglected alternatives contain value to the individual—value that had been obscured by the more salient goal. This theorized mechanism could help to explain why lay punishment judgments appear to be selectively responsive to cost prompts.

### Limitations and future directions

These results are not necessarily representative of U.S. voters and taxpayers as a whole. Compared to traditional undergraduate samples, the Mechanical Turk pool has been shown to be much more representative of the general population (see [29]), but it has not been normed for this purpose. Future research should attempt to replicate our findings using more representative samples.

The response modality used in this study has limited ecological validity since laypeople are usually not called upon to render sentencing recommendations. We made this choice because specific criminal case narratives provide a rich way of activating and revealing people’s moral attitudes. Even so, future research should attempt to reproduce our findings using more ecologically realistic tasks, such as voting on particular sentencing initiatives or economic games that contain real stakes.

Open questions remain about the types of costs and benefits most likely to influence punishment judgments. Experiment 1 showed that exposure to particular, mainly financial, costs of incarceration may be sufficient to mitigate punishment judgments, whereas Experiment 2 revealed that these effects could be carried by a mere emphasis of many other potential negative consequences of incarceration *that the participant generated*, such as family hardship, psychological damage, and even the criminogenic effects of detainment. This latter finding is consistent with remarks by professional judges when asked to explain their sentencing decision process in interview studies (e.g., [41]). It is plausible that the effect of such non-monetary factors on punishment recommendations could be even larger than the effect of direct financial costs alone. These non-monetary costs could also offer a potentially better test of possible demographic differences in sentencing attitudes (e.g., by political ideology or SES). Therefore, comparative tests of such consequences—teasing apart monetary costs, collateral opportunity costs, and other collateral consequences of incarceration—would greatly enhance research on punishment judgment formation.

It is possible that the reason the benefit manipulation in Experiment 1 did not increase sentencing recommendations is that the benefit manipulation failed to provide information that was not already believed by our participants, weakening the manipulation. Our study did not measure people’s prior expectations about the information presented, so we cannot address this interpretation directly. However, the fact that the effect replicated in Experiment 2 suggests that the difference between cost and benefit conditions is robust to the presence of information. Nonetheless, future research should control for differences in such expectations on the basis of careful pilot testing and targeted manipulation checks.

As with any vignette-based experiment, participants could have been motivated to respond in a way that confirms their preconceptions about the study hypotheses. Between-subjects designs were employed to reduce such demand characteristics, but may not eliminate them entirely. If such demand characteristics were operating in our experiments, then we should have observed confirmatory effects of both cost and benefit conditions, but the effects were specific to the cost condition. So, our results appear to be robust to such demands.

It is unclear why the marginal interaction with political ideology found in Experiment 1 did not replicate in the second experiment. One possibility is that the original effect was spurious, yet it is consistent with theoretical expectations that those endorsing a more conservative ideology would be less moved by cost considerations. Another possibility is that insufficient power was obtained in Experiment 2 to examine individual difference variables, given the smaller size of the main cost salience effect. Future research can address this question through the use of larger samples or by presenting opportunity costs that are likely to be more relevant to conservatives (e.g., tax rebate).

It is also noteworthy that self-reported retributive justifications did not attenuate the cost salience effect via an interaction. One interpretation of this null effect is that the cost salience effect is robust to variation in punitive motives. However, the punishment mean scores are consistent with the predictions of an interaction, so before accepting the null hypothesis, other alternative explanations must be ruled out, such as statistical power constraints and the possibility that our self-report measure of punishment justifications too weak a proxy for underlying punishment motives. This prospect raises the demand for a more implicit, behavioral measure of punishment justifications.

Finally, our conclusions are limited by the type of crime examined, namely drug trafficking. Previous research has found similar effects of cost information using cases of aggravated robbery and home invasion [20, 21]. We do not necessarily expect punishers to consider the costs of incarceration for the most serious crimes, such as murder, even after being prompted. Punishments for such crimes might be governed more strongly by deontological motivations that are insensitive to cost (e.g., retribution). Even so, an experimental test of this question will be a valuable contribution for future research.

These limitations notwithstanding, the present findings suggest the operation of a default benefits bias that inflates support for criminal punishment when corresponding cost information is not salient. This bias, it appears, can be lessened by efforts to make the decision costs more explicit, even without the introduction of new cost information. These results could help explain rising incarceration rates in the United States. They also have important implications for how cost and benefit information is delivered to voters and taxpayers. For instance, systematic efforts to balance information about the costs and benefits of correctional services on voting ballot materials could improve the internal consistency of voter support for sentencing measures. At the least, such materials could include information about the direct material costs of the punishment. Other types of costs could also be relevant, such as the punishment’s opportunity costs (e.g., funding withheld from offender reentry services) and/or the collateral consequences of the punishment (e.g., psychological damage, family hardship, employment barriers, criminogenic effects), but additional research is needed to test the relative contribution of these factors to sentencing attitudes.

If our findings represent a general property in human reasoning, we would expect to find evidence of sentencing cost neglect in prosecutors and judges too. Research has shown that punishment judgments by professional judges may depend on extra-legal, contextual factors [42] like pretrial publicity [43] and whether the judge's favorite football team just won or lost [44]. Studies have also shown that even subject-matter experts can neglect opportunity costs [10, 45]. Indeed, arguments for increasing transparency in the costs of incarceration have already been directed at judges and prosecutors. For instance, Miller [3] proposes possible legislative action to disclose such costs to judges in pre-sentencing reports. More broadly, several U.S. states, including Illinois, California, and Ohio, have begun to experiment with performance incentive funding, delivering grants and/or fines to counties that do and do not reduce their inmate populations [46]. However, additional research on the effects of cost disclosure on judicial sentencing attitudes is needed to confidently evaluate the need for and impacts of such practices.

Among non-experts, a clearer picture has begun to emerge, namely that making the costs of incarceration as salient as the benefits is likely to reduce support for punishment, at least for less serious crimes. Ultimately, understanding how such cognitive factors might facilitate more consistent and authentic punishment judgments is a worthy enterprise.

## Supporting information

S1 Appendix Experiment 2: Vignette and manipulations. (DOCX)
