# Supplementary material for: Slippery scales: Cost prompts, but not benefit prompts, modulate sentencing recommendations in laypeople
Source: PLoS One. 2020 Jul 31;15(7):e0236764. doi: 10.1371/journal.pone.0236764 (PMC7394432; doi:10.1371/journal.pone.0236764)
Supplement: S1 Appendix — (DOCX) [file pone.0236764.s001.docx]

# **S1 Appendix**

**Experiment 2: Vignette and manipulations**

Joseph Campbell, a high school dropout, was arrested at a party for allegedly selling 50 grams of methamphetamine. Joseph was charged and tried as a juvenile for felony drug trafficking. The evidence at trial, which included testimony from an undercover police officer and two other witnesses, showed convincingly that he exchanged the methamphetamine for $3,000 in cash. Joseph is 17-years-old, has a spotty employment record, and a history of drug addiction. He has one prior conviction for possession of methamphetamine. In your jurisdiction, methamphetamine sales carries a maximum sentence of 4 years in a secure juvenile detention center.

[COST STATEMENT:] Before you decide the defendant's sentence, you read a newly released government report. The report found that "prison sentences produce many negative consequences for the community." Please list three examples of possible negative consequences of incarcerating this defendant:

[BENEFIT STATEMENT:] Before you decide the defendant's sentence, you read a newly released government report. The report found that "prison sentences produce many positive benefits for the community." Please list three examples of possible positive benefits of incarcerating this defendant:

[CONTROL STATEMENT:] Before you decide the defendant's sentence, you read a newspaper containing a short puzzle. Please list three examples of words in the English language whose third letter is 'K':
